# Pediatric liver transplantation and COVID-19: a case report

**DOI:** 10.1186/s12893-020-00878-6

**Published:** 2020-10-06

**Authors:** Hamed Nikoupour, Kourosh Kazemi, Peyman Arasteh, Saba Ghazimoghadam, Hesameddin Eghlimi, Naghi Dara, Siavash Gholami, Saman Nikeghbalian

**Affiliations:** 1 Shiraz Transplant Center, Abu Ali Sina Hospital, Shiraz University of Medical Sciences, Shiraz, Iran; 2 Hepatology and Nutrition Research Center, Research Institute for Children’s Health, Shahid Beheshti University of Medical Sciences, Tehran, Iran

**Keywords:** COVID-19, liver, Transplantation, Pediatric

## Abstract

**Background:**

Immunosuppressed patients, including individuals with organ transplantation, have been among susceptible groups with regard to COVID-19, on the other hand pediatric patients more commonly undergo a mild clinical course after acquiring COVID-19. To the best of the authors knowledge, to this date very little data exists on COVID-19 in a pediatric patient with liver transplantation.

**Case presentation:**

We report a three year-old boy who had liver transplantation at 18 months old. He was admitted due to dyspnea with impression of acute respiratory distress syndrome and was then transferred to the intensive care unit. Chest X-ray at admission showed bilateral infiltration. Vancomycin, meropenem, azithromycin, voriconazole and co-trimoxazole were started from the first day of admission. On day 4 of admission, with suspicion of COVID-19, hydroxychloroquine, lopinavir/ritonavir and oseltamivir were added to the antibiotic regimen. PCR was positive for COVID-19. The patient developed multi-organ failure and died on day 6 of admission.

**Conclusions:**

For pediatric patients with organ transplantations, extreme caution should be taken, to limit and prevent their contact with COVID-19 during the outbreak, as these patients are highly susceptible to severe forms of the disease.

## Background

Immunosuppression is the main risk factor for infections which is an important cause of mortality and morbidity after liver transplantation (LT) in children. Bacterial and fungal organisms are the most common causes of infection in the first month after LT. After this duration, community-acquired viruses are the most common infections associated with chronic graft dysfunction (especially after 6 months). Cytomegalovirus and Epstein-Barr virus are the leading viruses which cause infection after the first month of LT [1, 2].

Currently, an outbreak related to a novel Coronavirus known as COVID-19 has become an international concern. LT recipients are among the most vulnerable groups with increased risk of infection but to this date there has been no report of COVID-19 in a pediatric patient with liver transplantation. Here we report a case of a pediatric patient who had LT and acquired COVID-19.

## Case presentation

A 3 year old boy, was admitted due to dyspnea in February, 2020. He presented with weakness, malaise, anorexia, severe dry cough, tachypnea and respiratory distress from 4 days prior to his admission.

In his past medical history the patient was premature and had liver cirrhosis due to biliary atresia. He underwent a living donor partial organ LT at 18 months old. Since then he received immunosuppressive medication which included prednisolone 5 mg daily and tacrolimus 2 mg daily.

At his last admission the patient’s mother mentioned a history of upper respiratory tract infection from 1 week ago that did not improve with regular medical therapies. On arrival, he had respiratory distress and decreased O2 saturation and tachypnea. He was admitted with impression of acute respiratory distress syndrome (ARDS) and was transferred to the pediatric intensive care unit immediately.

On examination, he was irritable, ill and toxic with a respiratory rate of 38–40/min, heart rate of 110/min, O2 saturation of 90–91% with a normal blood pressure and was afebrile. He was alert and orient with a Glasgow coma scale (GCS) of 15. His throat was congested without tonsillar exudation. In lung auscultation, harsh breath sounds were heard. Other physical examinations were normal.

His blood work results on admission were as follows: white blood cell (WBC) count of 12.8*10^3^/μl (with a neutrophil count of 10,880 and a lymphocyte count of 1024); red blood cell count, 4.6*10^6^/μl; platelet cell count, 187*10^3^/μl; hemoglobin, 13.8 g/dl; C-reactive protein (CRP), 102 mg/dl; erythrocyte sedimentation rate (ESR), 56 mm/h; sodium, 141 meq/L; potassium, 4.5 meq/L; magnesium, 1.9 mg/dl; blood urea creatinine (BUN), 22.3 mg/dl; creatinine, 0.6 mg/dl; glucose, 88 mg/dl; lactate dehydrogenase, 1277 U/L. His liver function profile was as follows: alanine transaminase (ALT), 28 U/L; aspartate transaminase (AST), 50 U/L; alkaline phosphatase (Alk-ph), 162 U/L; albumin, 2.7 g/dl; prothrombin time (PT), 16 s; partial thromboplastin time (PTT), 28 s; international normalized ratio (INR), 1.7. Patient’s blood tacrolimus level at final visitation was 10 ng/mL.

Vancomycin (50 mg daily), meropenem (90 mg daily), azithromycin (15 mg daily), voriconazole (50 mg daily) and co-trimoxazole (60 mg daily) were started from the first day of admission. About 48 h after hospitalization, the patient became unresponsive to continuous positive airway pressure (CPAP) and he was intubated. After which prednisolone and tacrolimus were discontinued.

During the third day of admission, his liver enzymes started to rise (ALT, 337 U/L; AST, 377 U/L). He developed acute kidney injury and BUN and creatinine increased up to 98 mg/dl and 2.5 mg/dl, respectively. Blood cultures were negative for growth of any microorganisms.

Chest x-ray on admission showed bilateral infiltration and on day 4 of admission it became a white lung. With suspicion of COVID-19, hydroxychloroquine (15 mg daily), lopinavir/ritonavir (100 mg daily) and oseltamivir (30 mg daily) were added to the antibiotic regimen. Due to the outbreak of COVID-19, a nasopharyngeal swab was taken and sent for real time polymerase chain reaction (RT-PCR) which was positive.

During the hospital course, the patient developed multi organ failure which included renal failure, liver failure and heart failure. On day 6 of admission, the patient developed excessive bleeding from the nose and mouth. Following which, cardiorespiratory arrest occurred and after 45 min of CPR the patient died 6 days after hospital admission. His laboratory tests on day 5 were as follows: WBC count, 10.8 × 10^3^/μl (with a neutrophil count of 9190 and a lymphocyte count of 702); RBC count, 4.6 × 10^6^/μl; platelet cell count, 165 × 10^3^/μl; hemoglobin, 10.8 g/dl; sodium, 146 meq/L; potassium, 5.1 meq/L; magnesium, 2.8 mg/dl; BUN, 92 mg/dl; creatinine, 2.3 mg/dl; glucose, 114 mg/dl; ALT, 4000 U/L; AST, 3000 U/L; Alk-Ph, 160 U/L; albumin, 3.3; total bilirubin, 0.2 mg/dl; direct bilirubin, 0.1 mg/dl; CRP, 109 mg/dl; ESR, 61 mm/h; PT, 16 s; PTT seconds, 28; INR, 1.7.

## Discussion and conclusion

Many transplant recipients have lymphopenia as a result of some of their medications. Coronavirus is among RNA viruses which can cause community-acquired respiratory virus (CARV) infections. Due to the immunity alterations in solid organ recipients, lower respiratory tract involvement caused by CARVs are higher [3]. From another aspect, children are more immunologically naïve. They have to use higher doses of immunosuppression in order to prevent rejection. Therefore, it is more likely that a more severe disease course and greater morbidity and mortality rate in pediatric transplant patients will be seen [4]. Infection rates after liver transplantation among the pediatric population are variable between studies. Nikeghbalian et al. [5] reported in-hospital infection rates in a large series of pediatric patients to be 9.4%, moreover infections constituted 35.2% of all causes of death in this population. Another experience from Korea reported the 6 month infection rate among pediatric patients to be 44.2% and similar to the previous report infections were the most common cause of death (50%) [6].

COVID-19 may lead to multiple organ dysfunction syndrome (ARDS, hepatic injury, acute kidney injury, acute cardiac injury) which result in death in severe cases [7]. We described a case of COVID-19 in a three year old solid organ recipient which presented with multi organ failure. According to a case series of 138 hospitalized patients with median age of 56 year old (42–68), elevated lactate dehydrogenase, prolonged thrombin time and many other laboratory abnormalities was seen in ICU patients in comparison to non-ICU patients [7]. These abnormalities was the same in our three year old patient. 

In another report, a 55 month-old girl with liver transplantation who recovered from COVID-19 was reported. She was infected with coronavirus disease 5 months after LT. It was concluded that using a high dose of immunosuppressant in a transplanted patient may not affect the severity of COVID-19, although immunosuppression has been related to more severe lower respiratory tract diseases in patients with COVID-19 [8].

Although more cases need to be studied, extreme caution should be taken for pediatric patients with organ transplantations, in here liver transplantation, to limit and prevent their contact with COVID-19 during the outbreak, as these patients are highly susceptible to severe forms of the disease.

## Data Availability

N/A.

## Abbreviations

LT: Liver transplantation
ARDS: Acute respiratory distress syndrome
GCS: Glasgow coma scale
WBC: White blood cell
ESR: Erythrocyte sedimentation rate
CRP: C-reactive protein
BUN: Blood urea creatinine
ALT: Alanine transaminase
AST: Aspartate transaminase
Alk-ph: Alkaline phosphatase
PT: Prothrombin time
PTT: Partial thromboplastin time
INR: International normalized ratio
CPAP: Continuous positive airway pressure
RT-PCR: Real time polymerase chain reaction
CARV: Community-acquired respiratory virus

## References

[1] Behairy B, Konsowa H, Zakaria H, Elsalam O, Sira M (2015). Infection after pediatric living related liver transplantation: timing, Types and Risk Factors. Int J Transplant Res Med.

[2] Green M, Michaels MG (2012). Infections in pediatric solid organ transplant recipients. J Pediatr Infect Dis Soc.

[3] Manuel O, López-Medrano F, Kaiser L, Welte T, Carratala J, Cordero E (2014). Influenza and other respiratory virus infections in solid organ transplant recipients. Clin Microbiol Infect.

[4] Michaels MG, Green M (2010). Infections in pediatric transplant recipients: not just small adults. Infect Dis Clin N Am.

[5] Nikeghbalian S, Malekhosseini SA, Kazemi K, Arasteh P, Eghlimi H, Shamsaeefar A, et al. The largest single center report on pediatric liver transplantation: experiences and lessons learned. Ann Surg. 2020. Publish Ahead of Print. 10.1097/SLA.0000000000004047 32541224

[6] Kim JE, Oh SH, Kim KM, Choi BH, Kim DY, Cho HR (2010). Infections after living donor liver transplantation in children. J Korean Med Sci.

[7] Wang D, Hu B, Hu C, Zhu F, Liu X, Zhang J (2020). Clinical characteristics of 138 hospitalized patients with 2019 novel coronavirus–infected pneumonia in Wuhan.

[8] Morand A, Roquelaure B, Colson P, Amrane S, Bosdure E, Raoult D (2020). Child with liver transplant recovers from COVID-19 infection. A case report. Archives de Pédiatrie.

